# Risk factors for failure of hydrostatic reduction of intussusception in pediatric patients: A retrospective study

**DOI:** 10.1097/MD.0000000000013826

**Published:** 2019-01-04

**Authors:** Xie Xiaolong, Wu Yang, Wang Qi, Zhao Yiyang, Xiang Bo

**Affiliations:** Department of Pediatric Surgery, West China Hospital, Sichuan University, China.

**Keywords:** failure of hydrostatic reduction, Intussusception, Pediatrics, risk factors

## Abstract

The aim of this current study was to explore the risk factors associated with failure of hydrostatic reduction of intussusception in pediatric patients.

Patients with intussusception treated with hydrostatic reduction from January 2010 to December 2016 were retrospectively analyzed. Candidates for inclusion in the study were children from 0 to 18 who were diagnosed with intussusception and treated with hydrostatic reduction. We excluded the patients who had contraindications for hydrostatic reduction, which included peritonitis, perforation signs, and non-responsive shock that required surgery. The data collected included: demographic data (sex, age, and bodyweight), symptoms (vomiting, abdominal pain, rectal bleeding, diarrhea, distention, constipation, and duration of symptoms), signs (temperature, palpable mass, and location of the mass), and other investigations (white blood cell counts, neutrophils, electrolytes, and ultrasound findings).

The risk factors for failure of hydrostatic reduction of intussusception were analyzed using the univariable analysis and the multivariable analysis. In the univariable model, the significant risk factors for failure of hydrostatic reduction of intussusception analyzed were age, bodyweight, duration of symptoms, rectal bleeding, constipation, palpable abdominal mass, poor prognosis signs on ultrasound scans and location of mass (the *P* value for each parameter are stated in Table 1

**Table 1:**
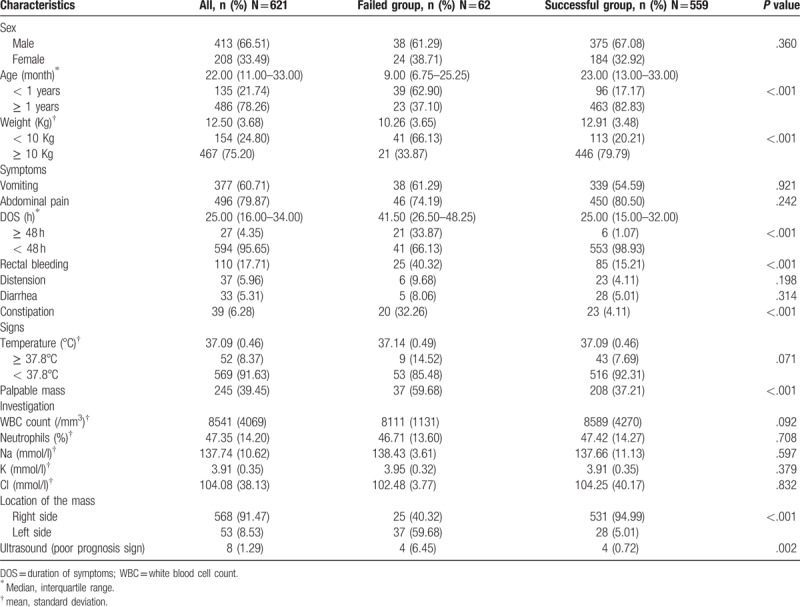
Baseline characteristics of the study population and univariate comparison of failed versus successful group.

). After the multivariable analysis was done, we found that the significant risk factors for failure of hydrostatic reduction of intussusception were an age of under 1-year-old (OR = 3.915, *P* = .027), duration of symptoms more than or equal to 48 h (OR = 0.056, *P* < .001), rectal bleeding (OR = 0.283, *P* = .003), constipation (OR = 0.086, *P* < .001), palpable abdominal mass (OR = 0.370, *P* = .010), and location of mass (left over right side) (OR = 13.782, *P* < .001).

Baseline characteristics of the study population and univariate comparison of failed versus successful group.

Our study found that an age of under 1-year-old, a duration of symptoms of more than or equal to 48 h, rectal bleeding, constipation, palpable abdominal mass and location of mass (left over right side) were risk factors for the failure of hydrostatic reduction of intussusception.

## 1 Introduction

Intussusception was a common cause of bowel obstruction and lower gastrointestinal bleeding in infants and children with an incidence of 1 in 4 in the year 2000.^[1]^ Intussusception is defined as the invagination of 1 segment of the intestine into part of the distal intestine. Delayed diagnosis and treatment may lead to bowel necrosis or even death.

There are 2 main kinds of intussusception: idiopathic and secondary to a pathologic lead point. Most of the cases (about 90%) are idiopathic,^[2]^ which means there is no obvious cause other than lymphoid hyperplasia of the terminal ileum, however in some cases invagination is secondarily induced by an identifiable cause (pathological lead point; PLP).

The diagnosis of intussusception, according to the clinical case definition for the diagnosis of acute intussusception proposed by the Brighton Collaboration Intussusception Working Group, can be determined by ultrasound with 100% accuracy by an experienced examiner.^[3]^ Currently, treatment modalities for intussusception include both non-operative and operative procedures. A non-operative procedure will likely be performed if no contraindications are present, which include: signs of peritonitis, perforation and a hemodynamically unstable patient in spite of adequate resuscitation.^[4–7]^ Operative procedures will be given when non-operative treatment is contraindicated or has failed.

The oldest and most widespread method is hydrostatic reduction with barium under fluoroscopic monitoring. In 1986, Guo JZ et al^[8]^ made an intussusception study in The People's Republic of China consisting of 6396 cases over a 13-year period which were successfully reduced by pneumatic reduction under fluoroscopic monitoring, resulting in a success rate of 95%. Since then, pneumatic reduction has increased worldwide and barium is no longer recommended. However, the use of the pneumatic reduction technique under fluoroscopy will expose children with intussusception to radiation and so an alternative technique often used is ultrasound-guided hydrostatic reduction with normal saline which can avoid radiation. Our center conducted a randomized trial of pneumatic reduction versus hydrostatic reduction for intussusception in pediatric patients which showed the success rate of hydrostatic reduction was 96.77%.^[9]^

Hydrostatic reduction failure is defined as intussusception that could not be reduced using an ultrasound-guide with normal saline. Although the prevalence and characteristics of intussusception are well established, little is known about failure of hydrostatic reduction of intussusception. The aim of this current study was to explore the risk factors associated with the failure of hydrostatic reduction of intussusception in pediatric patients.

## 2 Methods

This retrospective cohort study was approved by the ethics committees of West China Hospital of Sichuan University. Due to the retrospective nature of this study, our committee waived the need for patient consent. Between January 2010 and December 2016, patients with intussusception treated with hydrostatic reduction were retrospectively analyzed. The data was collected from patient charts and electronic medical records of the patients with intussusception (ICD-10 code K56.1) in West China Hospital of Sichuan University.

Candidates for inclusion in the study were children from 0 to 18 who were diagnosed with intussusception and treated with hydrostatic reduction. The diagnosis of intussusception was determined using an ultrasound conducted by an experienced examiner according to the clinical guidelines for the diagnosis of acute intussusception. We excluded the patients who had contraindications for hydrostatic reduction, which included peritonitis, perforation signs, and non-responsive shock that required surgery. The data collected included demographic data (sex, age, and body weight), symptoms (vomiting, abdominal pain, rectal bleeding, diarrhea, distention, constipation, and duration of symptoms), signs (temperature, palpable mass, and location of the mass), and other investigations (white blood cell counts, neutrophils, electrolytes, and ultrasound findings). The ultrasounds showed poor prognostic signs such as thick peripheral hypoechoic rim, free intraperitoneum fluid, fluid trapped within intussusception, enlarged lymph node in intussusception, and absence of blood flow in the intussusception.

**Figure 1:**
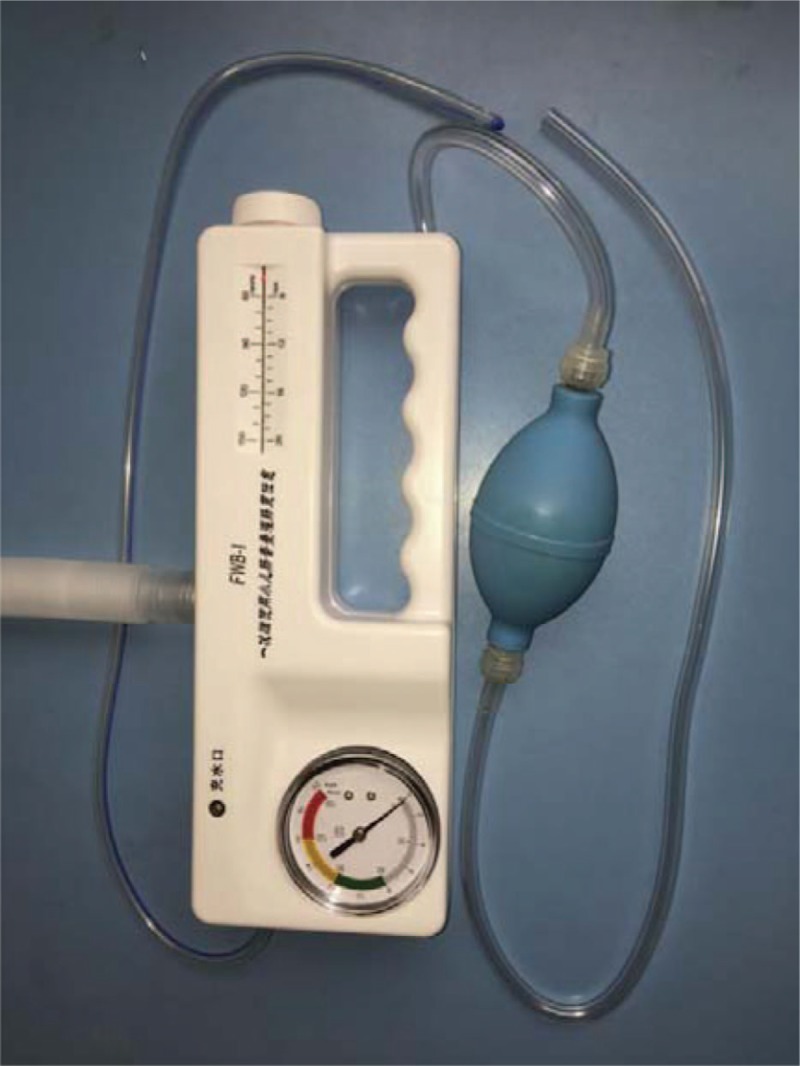
Photo of the apparatus of hydrostatic reduction.

The hydrostatic reduction was performed by a pediatric surgeon under ultrasound guidance with a 5 to 10 MHz transducer. A Foley catheter was inserted via the anus of the patient and the buttock was taped to prevent normal saline leakage. We used the pressure enema from 100 to 120 cmH2O in 3 separate attempts, each lasting 3 min. All patients received continuous pressure with the assistance of a balloon (Fig. 1). Sedation drugs were given according to the hospital sedation guidelines. The success of reduction was determined in 2 ways. Firstly, it was determined by the disappearance of intussusception and the visualization of the normal saline from the cecum to the ileum through the ileocecal valve or normal saline-distended ileum. Secondly, the disappearance of intussusception after reduction by ultrasound examination also determined the success. Failed reduction was defined as a remaining intussusception mass where normal saline could not pass from the cecum to the ileum through the ileocecal valve after the reduction procedure. In this case, an ultrasound was performed again to confirm the failure of the reduction. The patients were then divided into 2 groups: the failed group and the successful group.

### 2.1 Statistical analysis

Data was entered into the database by 1 author and checked by one of the study's other authors. Statistical analysis was performed using SPSS version 23.0. The descriptive data was reported in number and percentage form for categorical data, and mean and standard deviation or median and interquartile range for continuous data. Differences were evaluated using Student's *t* test for continuous parametric data, the Wilcoxon test for continuous nonparametric data and Pearson chi-squared test for noncontinuous data. Logistic regression was performed to identify independent risk factors. A *P* value of less than .05 was considered statistically significant.

## 3 Results

**Figure 2:**
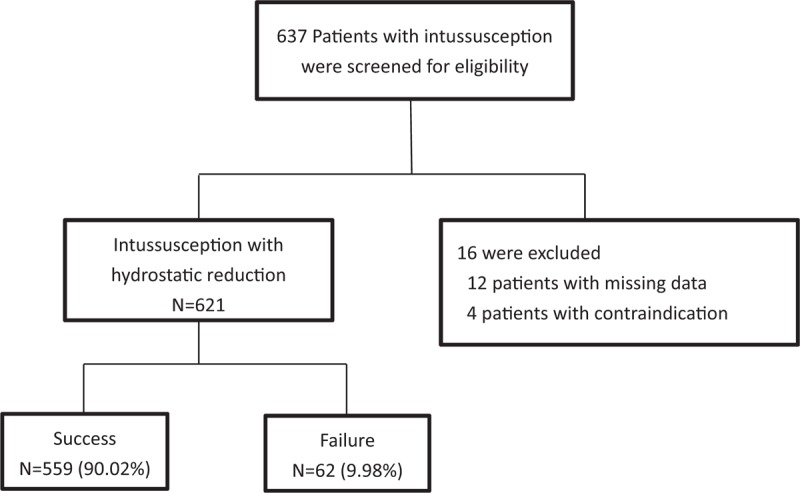
Study flow of hydrostatic reduction for intussusception.

A total of 637 patients with intussusception were treated with hydrostatic reduction at our institution over a 7-year period (Fig. 2). According to the retrospective study, missing data elements were identified in 11 records which were thus excluded and 6 additional patients were also excluded due to the contraindications after the diagnosis. A total of 621 episodes of intussusception were collected for final analysis. A total of 62 (9.98%) patients suffered failed reduction.

The specifics and outcomes of the study's subjects are described in Table 1. The male to female ratio was 2:1. The median age of the patient was 22 months with a mean weight of 12.50 kg. The most common symptoms were vomiting, abdominal pain, and rectal bleeding (60.71%, 79.87%, and 17.71%, respectively). Diarrhea was found in 5.31% and constipation was found in 6.28% of the patients. A palpable abdominal mass and abdominal distension were observed in 39.45% and 5.96% of the patients respectively. The median duration of symptoms before presentation was 25 h. The most common location of the palpable mass was on the right side of the abdomen and this was found in 91.47% of the patients.

The risk factors for failure of hydrostatic reduction of intussusception were analyzed using the univariable analysis (Table 1) and the multivariable analysis (Table 2). In the univariable model, the significant risk factors for failure of hydrostatic reduction of intussusception analyzed were age, body weight, duration of symptoms, rectal bleeding, constipation, palpable abdominal mass, poor prognosis signs on ultrasound scans and location of mass (*P* value for each parameter are stated in Table 1). After the multivariable analysis was done, we found that the significant risk factors for failure of hydrostatic reduction of intussusception were an age of under 1-year-old (OR = 3.915, *P* = .027), duration of symptoms of more than or equal to 48 h (OR = 0.056, *P* < .001), rectal bleeding (OR = 0.283, *P* = .003), constipation (OR = 0.086, *P* < .001), palpable abdominal mass (OR = 0.370, *P* = .010), and location of mass (left over right side) (OR = 13.782, *P* < .001).

**Table 2:**
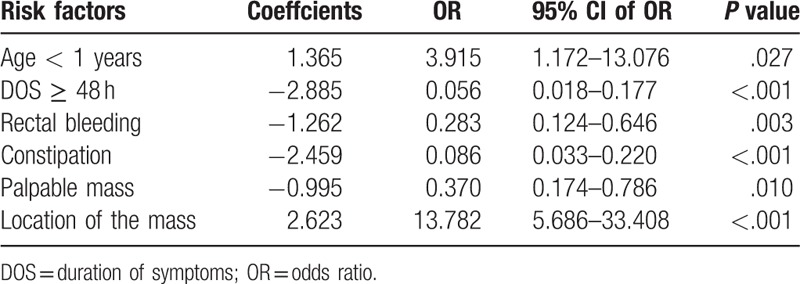
Multivariate analysis of risk factors.

Afterward, all patients with failed reduction were operated on. The operative findings of the intussusception patients with failed reduction are shown in Table 3. Of the 62 failed reduction patients, 39 cases (62.90%) were idiopathic intussusception while 23 cases (37.10%) were induced using pathological lead point. The pathologic leading points reported were intestinal polyp, Michael diverticulum and intestinal angioma. The ileocolic type (93.55%) was the most common. All 62 patients were eventually discharged and made successful recoveries.

**Table 3:**
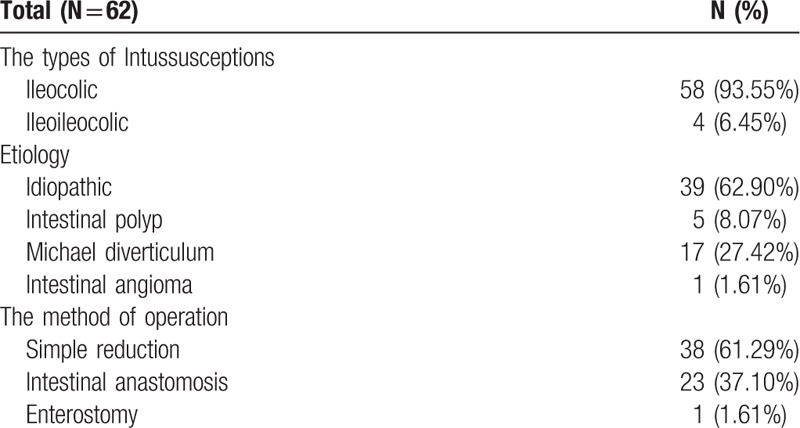
Analysis of the failed reduction of intussusception.

## 4 Discussion

This study was the second in a series of studies on intussusception conducted in our institutions. The first study reported on a randomized trial of pneumatic reduction versus hydrostatic reduction for intussusception in pediatric patients. We found that the success rate for hydrostatic reduction was 96.77%.^[9]^ Therefore, this study was created to identify the factors that lead to failed reduction. The significant risk factors identified in our study were found to be an age of under 1-year-old, duration of symptoms of more than or equal to 48 h, rectal bleeding, constipation, palpable abdominal mass and location of mass (left over right side).

Other similar studies in the past also pointed to the age factor in patients as a risk for failed reduction. Fallon et al^[10]^ and Tota-Maharaj et al^[11]^ found that an age of under 1-year-old was significantly associated with failed reduction. In our study as well, an age of less than 1-year-old was also significantly associated with failed reduction. This result may be attributed to the small caliber of the small bowel found in young children so as a result the intussusception was difficult to reduce. The reason why the age of under 1-year-old and weight below 10 kg are statistically significant in univariate analysis while weight above 10 kg is not thought to be the risk factor leading to failure of hydrostatic reduction of intussusception in multivariate analysis may be that the predictive factor of the body weight is related to the age.

The duration of symptoms associated with failed reduction remains controversial. The symptom complex of vomiting, abdominal pain, and passage of watery and bloody stool may mimic gastroenteritis, malaria, and other causes of acute abdomen in children. This often leads to initial misdiagnosis and late referral. Wong et al^[12]^ found that a mean duration of symptoms of 2.3 days did not affect success rate of the reduction in Hong Kong. Reijnen et al^[13]^ stated that a duration of symptoms of 48 h was a significant predictor of failure of hydrostatic reduction. In our study, duration of symptoms equal or more than 48 h was significantly associated with failed reduction. It may be that a long duration of symptoms before treatment directly leads to a loss of intestinal viability.

Constipation is one of the symptoms of intussusception. From the previous study, constipation was found to be a symptom helpful in the diagnosis of intussusception but not a statistically significant predictor of failed reduction as found in our study. Constipation and dry stool may reduce intestinal volume and decrease the pressure of hydrostatic reduction.

Rectal bleeding and abdominal mass are the 2 most common and traditional signs of intussusception. He et al^[14]^ found that rectal bleeding was a predictor of failed reduction as in our study. Palpable abdominal mass was also a significant factor associated with failed reduction in our study and in the study of Wong et al^[12]^. He et al also found that the intussusception located on the left side of the abdomen was significantly associated with a lower success rate of reduction. Most cases of intussusception were of the ileocolic type. The location of the mass represents the length of intussusception. In our study, a mass located on the left side of abdomen was significantly associated with failed reduction.

There are several limitations to our study. First, the sample size was small because of the low risk of failure. Second, there were some possibly unknown risk factors that we were unable to measure. Finally, the number of studies reporting the risk factors for failure of hydrostatic reduction of intussusception was limited. Despite these limitations, our study improves the understanding of the risk factors for failure of hydrostatic reduction of intussusception in pediatric patients.

## 5 Conclusion

Our study found that an age of under 1-year-old, a duration of symptoms more than or equal to 48 h, rectal bleeding, constipation, palpable abdominal mass and location of mass (left over right side) were risk factors for failure of hydrostatic reduction of intussusception.

## Author contributions

**Conceptualization:** Zhao yiyang, Xiang Bo.

**Data curation:** Wu yang, Wang qi, Xiang Bo.

**Formal analysis:** Wang qi, Zhao yiyang.

**Investigation:** Xie xiaolong, Xiang Bo.

**Methodology:** Wu yang, Xiang Bo.

**Project administration:** Zhao yiyang, Xiang Bo.

**Resources:** Xie xiaolong, Wu yang, Xiang Bo.

**Software:** Wu yang, Wang qi.

**Visualization:** Xiang Bo.

**Writing – original draft:** Xie xiaolong.

**Writing – review & editing:** Xie xiaolong.
